# Assessing the Long-Term Performance of Adhesive Joints in Space Structures during Interplanetary Exploration

**DOI:** 10.3390/ma16144978

**Published:** 2023-07-13

**Authors:** Gabin Charpentier, Ugo Lafont, Sofia Teixeira de Freitas

**Affiliations:** 1 Department of Aerospace Structures and Materials, Faculty of Aerospace Engineering, Delft University of Technology, Kluyverweg 1, 2629 HS Delft, The Netherlands; gabincharpentier@gmail.com; 2 ISAE ENSMA, Téléport 2, 1 Avenue Clément Ader, 86360 Chasseneuil-du-Poitou, France; 3 European Space Agency, Keplerlaan 1, P.O. Box 299, 2200 AG Noordwijk, The Netherlands; ugo.lafont@esa.int

**Keywords:** adhesives, space environment, aging

## Abstract

Spacecraft experience minimal mechanical loads in space, but with the development of reusable spacecraft for interplanetary exploration and repeated landings, structures will be subjected to increased mechanical stress. The impact of the space environment on the aging of adhesive materials used in space structures over long-term applications is not well understood. This study investigates two commonly used adhesives in spacecraft assembly, namely Scotch-Weld™ EC-2216 and Scotch-Weld™ EC-9323-2, under two aging conditions: (1) high-energy electron irradiation using a Van de Graaf accelerator, and (2) thermal vacuum cycling. The research evaluates the evolution of intrinsic adhesive properties and adhesion to CFRP (carbon fiber-reinforced polymer) and aluminum adherents before and after exposure to these environmental conditions through tensile tests, peel tests, double-cantilever beam (DCB) tests, and dynamic mechanical analysis (DMA).

## 1. Introduction

Spacecraft and satellites experience minimal mechanical loads in space. However, with the advent of reusable spacecraft designed for interplanetary exploration and repeated landings on celestial bodies like the Moon, Mars, or asteroids [1,2], the structures will face more repetitive mechanical loads even after extended periods of exposure to the space environment.

In such conditions, the intrinsic properties of materials will undergo changes. One of the initial effects of space vacuum exposure is outgassing, where organic components release molecules into space [3,4,5]. However, minimizing and controlling outgassing are primarily driven by the need to avoid molecular contaminants that could be released and later re-adsorbed by sensitive spacecraft surfaces, such as optics. Outgassing can be mitigated before launch using materials with very low outgassing characteristics and implementing pre-launch bakeout strategies. At this stage, the integrity of adhesives remains unaffected by outgassing and can be disregarded as a relevant parameter. Radiation is also a significant factor in the space environment. It encompasses a wide spectrum of light wavelengths, including energetic photons (UV-rays, X-rays, γ-rays) from the solar flux and charged particles (electrons, protons, nucleons) trapped by electromagnetic fields like the Van Allen belt around the Earth [6]. Different types of radiation can have varying effects on the mechanical properties of polymers. Radiation can cause molecular chain scission, recombination, cross-linking, deformation, embrittlement, and discoloration, which can affect the mechanical integrity of the polymer [7,8]. Another environmental factor in low Earth orbit is Atomic Oxygen (ATOX), which can induce material degradation through etching and pitting, although it is generally not a concern for adhesives due to their protection from direct exposure [7], and it will not be considered in this study.

Operational temperature in the space environment can fluctuate along a wide range. This range depends on the mission scenario, the distance from the sun, and spacecraft exposure and can lead to variation from −200 °C to +200 °C for the most extreme mission scenario. In addition, it is important to mention that a change of temperature can occur very fast when a spacecraft passes from direct sun illumination to a sun-shaded position. In this respect, all missions present different thermal environments that drive the choice of material and the thermal management strategy. If not fully defined, material validation for space environments is subjected to a thermal cycling screening test from −100 °C to +100 °C as defined in ECSS-Q-ST-70-04C [9]. For space applications, adhesives are required to be resistant to such extremes [8,10]. Temperature gradients can cause thermal residual stresses at the bond line between materials with different coefficients of thermal expansion, potentially leading to stresses, failures, damage, delamination, and more.

Moreover, one of the major challenges related to adhesives is differentiating between adhesion and the material properties of the adhesive itself. Adhesion represents the force bonding a material to the adhesive, quantifiable through various adhesion mechanical tests [11,12] such as peel tests. The intrinsic material properties of the adhesive, on the other hand, are determined primarily through physical–chemical and mechanical tests specific to the adhesive material [13].

The choice of adherent is another crucial consideration for adhesives. In most spacecraft, composite materials like CFRP and lightweight metals like aluminum are commonly used [14,15]. Whereas there is ample literature on the mechanical parameters of adhesives in contact with these two types of surfaces both for unaged specimens and those subjected to hydrothermal aging [11,16,17,18,19], information on the durability of adhesion under space environments is relatively limited.

The durability of adhesive materials used in space structures under repeated launching loads and exposure to space conditions over extended periods (e.g., 10, 15, 20 years of exploration) remains unknown. Lifetime prediction is possible using activation energy and simple models, like model-free kinetics [20]; however, using such model for adhesive bond is still a challenge for space applications due to the many influencing parameters related to the space environment. The key question is to anticipate the degradation of the mechanical and physicochemical properties of adhesives after a specific number of years. The literature lacks previous studies on the aging of certain material properties under space conditions, particularly in terms of adhesion assessment using mechanical and physical testing. This research aims to determine the durability of adhesion-related properties under space environment conditions, including fracture toughness, peel load, and surface failure modes. In addition, material properties such as glass transition temperature, Young’s modulus, tensile strength, and strain at break is investigated.

This study examines the effects of thermal vacuum cycling and high-energy electron irradiation on the properties of two widely used adhesives in space applications: Scotch-Weld™ EC-2216 and Scotch-Weld™ EC-9323-2 (3M, Bracknell, UK). The adhesive behavior in composite-to-composite and aluminum-to-aluminum joints was evaluated using double-cantilever beam tests (DCB) and floating roller peel tests (FRPT), respectively. The evolution of adhesive intrinsic properties, such as tensile strength and thermo-mechanical behavior, was investigated through tensile tests and dynamic mechanical analysis (DMA).

## 2. Materials and Methods

### 2.1. Materials

Two types of paste adhesives were investigated: Scotch-Weld™ EC-2216 and Scotch-Weld™ EC-9323-2. These are epoxy-based, two-component adhesives that can be cured at room temperature or at 65 °C. The curing strategy will impact the T_g_. Table 1 lists the curing process used in this study (time and temperature) and the mixing ratio between the two parts. The materials were prepared and cured at 65 °C for 2 h as per the manufacturer’s data sheet. Both adhesives are space-qualified but are used in different applications [21]. The EC-2216 is used for bonding non-structural elements, and the EC-9323-2 is mainly used in structural applications.

To assess the adhesion to CFRP substrates, double-cantilever beam (DCB) specimens were produced using previously cured CFRP (secondary bonding). CFRP were prepared from a unidirectional pre-preg consisting of Hexply 8552 epoxy matrix in combination with AS4 carbon fiber (Hexcel Corporation, Stamford, CT, USA). To produce the two-rigid adherent of DCB samples, a lay-up of 8 plies (0°)s of approximately 1.6 mm was used. The laminate was cured for 120 min at 180 °C. Prior to bonding, the cured CFRP surfaces were prepared by means of abrasion with sandpaper and cleaned with acetone.

To assess the adhesion to aluminum, floating roller peel test specimens were produced using clad aluminim alloy 2024. The aluminum surfaces were pre-treated by grit blasting, coated with Sol-Gel 3M™ AC-130-2, and sprayed with 3M Scotch-Weld™ Primer 3901 (3M, Bracknell, UK).

### 2.2. Methods

For tensile tests, the experimental procedure was based on the standard test method for the tensile properties of plastics described in ASTM D638 [22]. Testing was carried out using a Zwick machine (Zwick, Ulm, Germany) equipped with a load cell of 1 kN. For EC-2216, the test speed was 5 mm·min^−1^, and the crosshead displacement and load were recorded at 5 s and 0.42 mm, respectively. For EC-9323-2, the test speed was reduced to 0.5 mm·min^−1^, and the crosshead displacement and load were recorded for all 5 s and 0.042 mm, respectively. This test speed reduction was to ensure similar data acquisition points before failure between the two adhesives (as EC-9323-2 is a more brittle than EC-2216). Displacement data were recorded using a Digital Image Correlation (DIC) setup.

The dynamic mechanical analysis (DMA) was performed following the experimental procedure of the standard test methods for glass transition temperature, loss modulus, and storage modulus described in ASTM D7028, E2254, and E2425 [23,24,25]. Testing was carried out using a TA Instrument RSA G2 machine (TA Instrument, New Castle, DE, USA) with a temperature range between −40 °C and 140 °C and a ramp rate of 2 °C·min^−1^. The test was performed in flexion mode (3-point bending).

For the floating roller peel test (FRPT), the experimental procedure was based on the standard test method described in ASTM D3167 [26]. Testing was carried out using a Zwick machine (Zwick, Ulm, Germany) coupled with a load cell of 1 kN. The testing speed was 125 mm·min^−1^.

For the double-cantilever beam (DCB), the test procedure followed the standard method of determining fracture toughness described in ASTM D5528 [27]. For this purpose, a Zwick machine (Zwick, Ulm, Germany) coupled with a load cell of 1 kN was used. The testing speed was 2 mm·min^−1^. The crack length propagation in the bond line was monitored using millimetric paper bonded to one side of the specimen and an optical camera.

### 2.3. Sample Preparation

**Figure 1:**
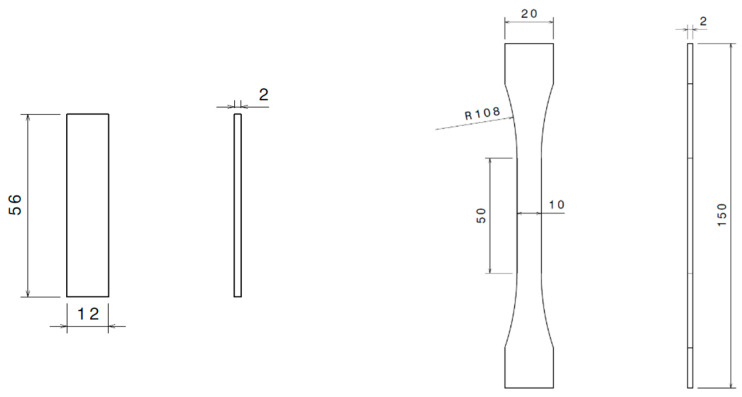
Sample shape and dimension for (**left**) DMA and (**right**) tensile testing (dimensions in mm).

Sample preparation was carried out in compliance with the adhesive bonding processes for spacecraft and launcher applications defined by ECSS-Q-ST-70-16C [28]. DMA and tensile test specimens were produced via machining of plates of pure adhesive material. These plates were produced by placing adhesive between two glass plates with a control thickness of 2 mm and cured in an oven (as per Table 1). Figure 1 shows the specimens dimensions for DMA specimens as described in ASTM E2254 and ASTM E2425 [24,25] and for tensile testing as described in ASTM D638 standard [22].

**Figure 2:**
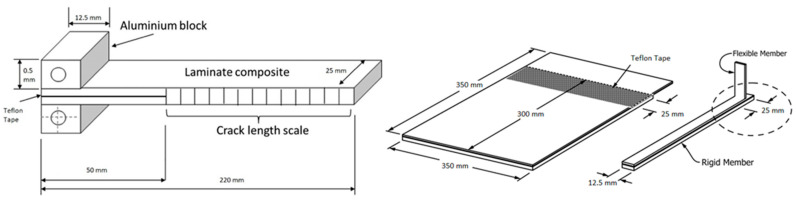
(**left**) Composite-to-composite DCB and (**right**) aluminum-to-aluminum FRPT specimen dimension.

Figure 2 shows the DCB specimens, FRPT specimens, and their corresponding dimensions. In the FRPT, the flexible aluminum sheets were 0.6 mm thick, and the rigid aluminum sheets were 1.6 mm thick. A Teflon tape was positioned at the first 75 mm of the flexible adherent length (25 mm for the opening crack). To control the adhesive bond line, 0.1% wt of glass beads of 250 microns in diameter were added to the paste adhesive mixture. The blend was then spread on the aluminum plates, which were then joined together using weights and cured in an oven (as per Table 1). The FRPT specimens’ dimensions were 12.5 mm wide and 350 mm long with a bond line thickness of 0.15 ± 0.05 mm based on the standard test method ASTM D3167 [26].

In the DCB test specimens, similar glass beads were added with the same ratio to the paste adhesive mixtures as for the floating roller peel test procedure and cured in an oven. DCB specimen dimensions were based on the standard test method ASTM D5528 [27] for mode I interlaminar fracture toughness of unidirectional fiber-reinforced polymer matrix composites. CFRP panels of 1.6 mm thickness were used to manufacture composite-to-composite DCB samples. Specimens were 25 × 220 mm with a 50 mm crack opening (materialized with a Teflon tape) and a bond line thickness of 0.17 ± 0.06 mm.

Both aluminum-to-aluminum (FRTP test) and composite-to-composite (DCB test) products were then machined to cut out specimens with the desire dimensions.

### 2.4. Space Environment Exposure

In order to mimic space environment conditions, two different types of space environments were considered in this study: thermal vacuum cycling and irradiation with high energetic electrons. The thermal vacuum cycling test was carried out at ESA-ESTEC (Noordwijk, The Netherlands) using a dedicated chamber for 25 cycles between −100 °C and +100 °C at 10^−6^ mbar. The period of each cycle was 4 h, and the total duration was about 100 h. Electron irradiation was performed at the Delft Reactor Institute (Delft, The Netherlands) using a Van de Graaf accelerator. Samples were subjected to a total ionizing dose of 1 MGy using an electron beam delivering 1.5 × 10^12^ cm^−2^·s^−1^ for 3.5 h.

A total of five specimens were tested per adhesive and configurated with the following conditions: (1) unaged/pristine, (2) after irradiation exposure, and (3) after thermal vacuum cycling.

## 3. Results and Discussion

### 3.1. Effect Space Environment on Adhesive Intrinsic Properties

In order to evaluate the aging effect on the adhesives, five parameters were derived from the experimental results: Young’s modulus, tensile strength and strain at break (tensile tests), glass transition temperature (DMA and DSC tests), and heat flow (DSC tests).

**Figure 3:**
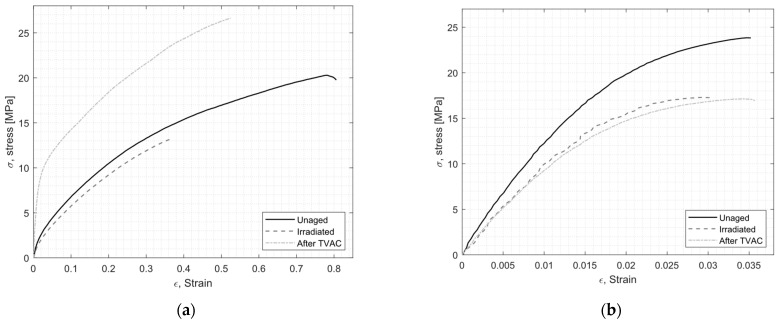
Typical tensile stress-strain of EC-2216 (**a**) and EC-9323-2 (**b**) as-produced (unaged), after irradiation, and after thermal vacuum aging cycling (TVAC).

Figure 3 shows the representative stress-strain curves obtained for both adhesives under the three conditions. The average values for Young’s modulus, tensile strength, and strain at break are given in Table 2.

For EC-2216 (Figure 3a), it can be noticed that behaviors were different after particle irradiation and TVAC. After particle irradiation, the average Young’s modulus dropped from about 240 MPa for a non-aged sample to about 120 MPa for an irradiated sample. On the contrary, after thermal vacuum aging cycling (TVAC), the average Young’s modulus increased to about 600 MPa. The stress-strain curve in its elastic zone, after TVAC, was much steeper and presented a larger elastic zone than for unaged material. This was also reflected in the tensile strength value that increased by almost 50% after TVAC, whereas it decreased by about 25% after irradiation. Finally, both types of aging had the same effect on the elongation at break by decreasing its value, therefore decreasing the ability of this adhesive to elongate prior to failure when subjected to tensile loading, even though the particle irradiation decreased it more than in the TVAC sample. It is clear for EC-2216 that irradiation led to a general decrease of the tensile properties whereas TVAC led to an overall increase. Such improvement after TVAC can be attributed to a post-curing effect occurring during the thermal cycling.

For EC-9323-2 (Figure 3b), the tensile behavior was similar after TVAC and particle irradiation. However, compared to the non-exposed samples, the modulus decreased by a factor 2 from 2.2 GPa to 1.1 GPa for the pristine sample and the sample exposed to irradiation and TVAC, respectively. The same trend was observed in the tensile strength, with decreases after both aging conditions. The elongation of EC-9323-2 was almost unaffected after aging. However, the fracture surface of the sample revealed the presence of small voids for EC-9323-2 for all samples that were not present for EC-2216. Even if such inhomogeneity could have a negative effect on the general tensile properties, the EC-9323-2 tensile test reached a plateau, as expected, with respect to the mechanical behavior of such material. However, the strain might be not fully representative and shall not be considered. This difference of homogeneity between the two types of adhesives was related to the preparation process. In this study, the mixing processes were identical for both adhesives and potentially not optimized. For space applications, each bonding procedure was qualified and validated, leading to a stable and reproducible process. This aspect was not the focus of this study, although it is clear that this has an impact on the optimum performance of the adhesive.

**Figure 4:**
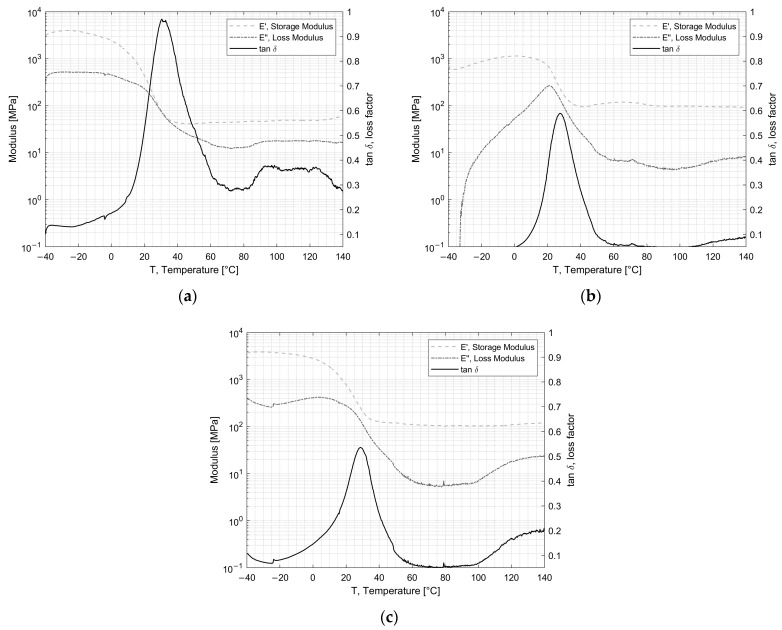
Evolution of the storage and loss modulus, tan δ for EC-2216: (**a**) unaged (**b**) after irradiation and (**c**) after TVAC.

**Figure 5:**
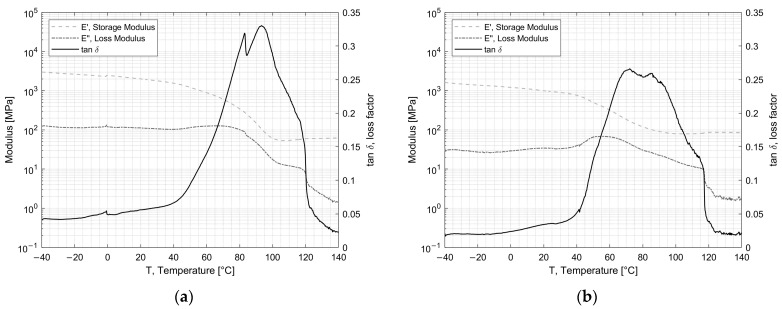

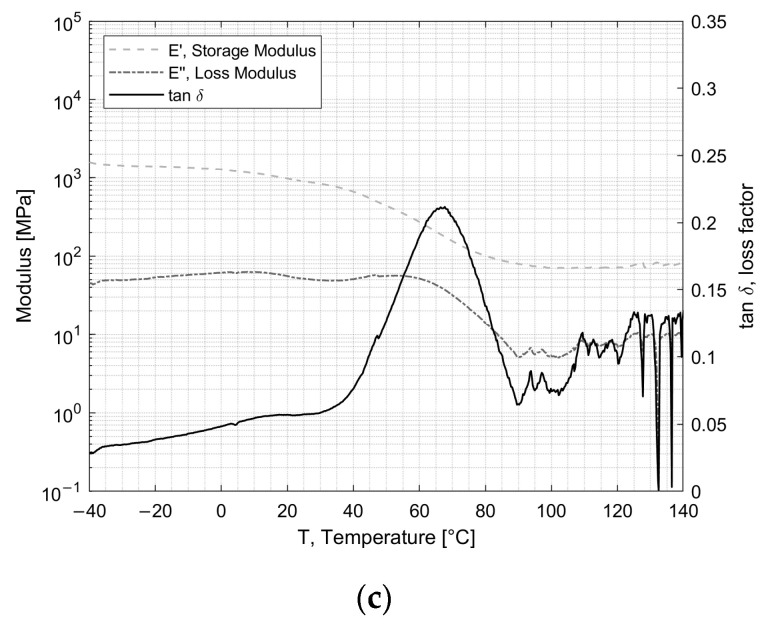
Evolution of the storage and loss modulus, tan δ for EC-9323-2: (**a**) unaged, (**b**) after irradiation and (**c**) after TVAC.

DMA (dynamical mechanical analysis) was performed to investigate the visco-elastic behavior and the glass transition temperature (T_g_). The elastic storage modulus (E’), which is proportional to the energy fully recovered per deformation cycle; the loss modulus (E”), which is proportional to the net energy dissipated in heat per cycle; and tan δ, the loss factor which represents the damping during dynamic deformation, were recorded. Figure 4 and Figure 5 show the results for the adhesives EC2216 and EC9393-2, respectively. The T_g_ was extracted at the temperature at which tan δ was maximum. T_g_ values are reported in Table 3.

For EC-2216 depicted in Figure 4, it can be seen that after irradiation or TVAC exposure, the T_g_ increased by 2 °C in comparison to the pristine/unaged material. This change was not very significant considering the standard deviation. However, the value of the maximum of tan δ, which was around 1 for unaged sample, dropped around 0.6 after TVAC and irradiation. This means that the ability of the adhesive to dissipate energy into heat after aging was lower. This could be explained by the average diminution of Loss Modulus with aging. Indeed, in Figure 4b, it can be seen that after irradiation over the temperature range from −40 °C to 20 °C, the loss modulus increased from almost zero to a peak at about 300 MPa, in contrast to the unaged sample, where it gradually decreased throughout the temperature range. After TVAC, the loss modulus followed the same pattern as for the unaged sample, except it increased significantly from 100 °C to 140 °C. These increases mean that over the relevant intervals, the EC-2216 experienced an increase in the net energy dissipated per cycle. The storage modulus, which is proportional to the total energy recovered per deformation cycle, does not change significantly after aging. It can also be noticed that the glassy state, the glass transition range, and the rubbery plateau did not change in a major way for the unaged and irradiated samples as they were, respectively, between −40 °C and 0 °C, between 0 °C and 60 °C, and between 60 °C and 100 °C. After TVAC (i.e., 25 cycles between −100 °C and 100 °C) the glass transition range was between −30 °C and 60 °C. Furthermore, the tan δ decreased between −40 °C and −30 °C, which suggests a flow region for lower temperatures.

For EC-9323-2 depicted in Figure 5, the glass transition zone extended from 40 °C to 120 °C for unaged and irradiated samples. After TVAC, this range was again modified, but this time, with a decrease from 40 °C to 90 °C. After this zone, there was no longer a rubbery plateau, as it was replaced by a zone of great instability created by TVAC, as shown in Figure 5c. Moreover, for this adhesive, the irradiation exposure did not change the general behavior of E’ and E”. The main change concerned the T_g_, as shown in Table 3. Indeed, initially situated around 91 °C, the latter dropped to 72 °C after irradiation and 68 °C after TVAC. As irradiation creates chain splitting and cross-linking which are two opposing phenomena, it was necessary to carry out additional tests to determine which of these phenomena predominated in the adhesives concerned and whether they had an impact on the T_g_.

### 3.2. Impact of Space Conditions on Adhesion Properties

**Figure 6:**
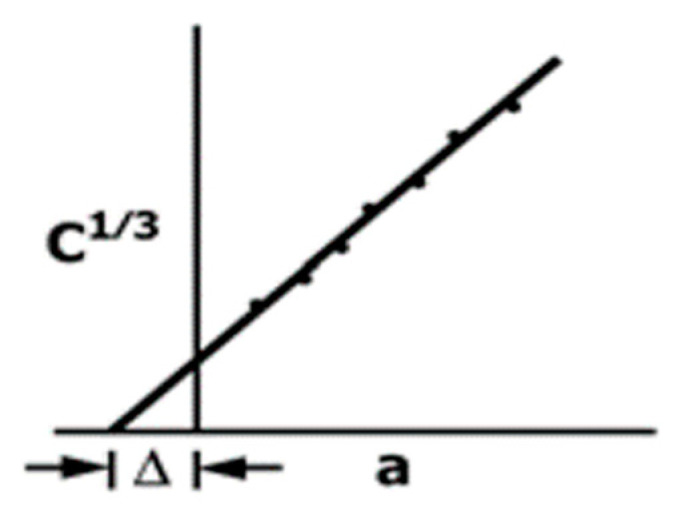
Δ is a factor for the modified beam theory (standard test method ASTM D5528).

For the DCB test, using DIC (Digital Image Correlation) periodic image recordings, the crack growth was obtained. With these values, the opening Mode I interlaminar fracture toughness $G_{I_{c}}$ was determined using Equation (1).

(1) $$G_{I_{c}}=\frac{3P\delta }{2b(a+\left|\Delta \right|)}$$

where *P* is the applied load, *a* is the crack length, *b* is the width of the DCB specimen, *δ* is the load point displacement, and Δ is a factor determined by generating a least-squares plot of the cube root of compliance as a function of the crack length using Equation (2) following the standard test method ASTM D5528 for the modified beam theory (see Figure 6).

(2) $${C\;}^{\frac{1}{3}}={\left(\frac{\delta }{P}\right)}^{\frac{1}{3}}$$

**Figure 7:**
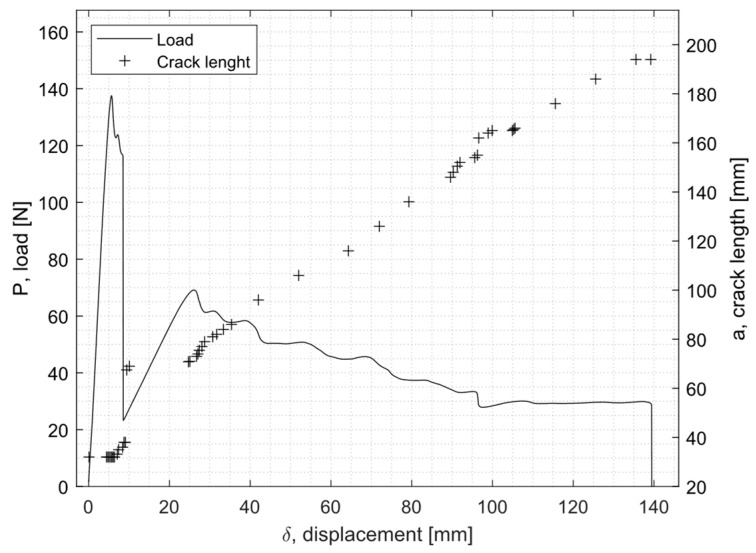
Example of the load displacement curve for the EC-2216 unaged DCB specimen.

**Figure 8:**
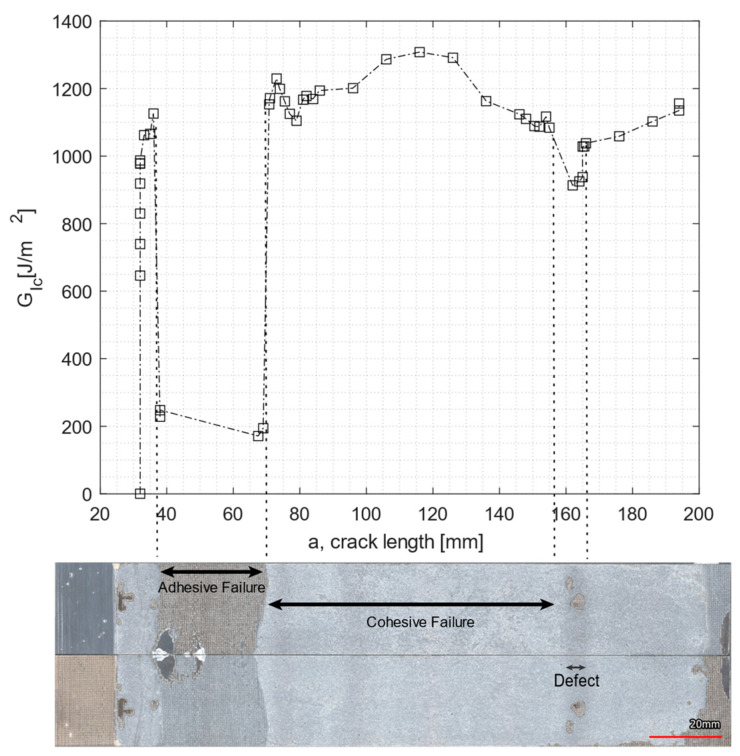
R-curve for the EC-2216 unaged DCB sample and the corresponding failure surface of both adherents.

Figure 7 shows an example of a load displacement curve as well as the crack growth for EC-2216. Figure 8 shows the evolution of the fracture toughness $G_{I_{c}}$ (R-curve) and the corresponding fracture surface of the specimen.

From Figure 8, it can be seen that a drop in the value of the fracture toughness corresponded to the fracture surface of an adhesive failure. In addition, a second drop in the $G_{I_{c}}$ occurred further on the crack length due to a change of failure mode created by a manufacturing defect, which also caused $G_{I_{c}}$ to drop.

**Figure 9:**
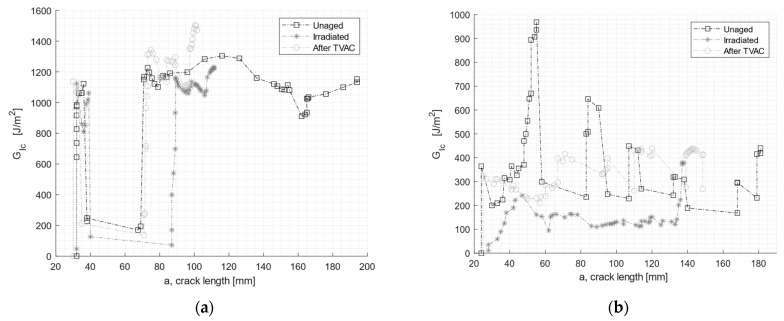
R-curves of the DCB test for unaged, after irradiation, and after TVAC for (**a**) EC-2216 and (**b**) EC-9323-2.

Figure 9 compares representative R-curves for unaged and aged specimens. Table 4 lists the average values of $G_{I_{c}}$ for both adhesives and tested aging conditions. In Figure 9a, the initial drop in the $G_{I_{c}}$ in the EC2216 adhesive was related to a region with adhesive failure, also present in the specimen shown in Figure 8 (same batch of specimens). In Figure 9b, $G_{I_{c}}$ values were quite unstable with peaks and lows. This was related with the significant manufacturing defects present in the specimens of this adhesive. This limited the representative results of the DCB test of EC 9323-2.

The two aging conditions affected the adhesives’ performance differently. For both adhesives, the average value of the fracture toughness decreased after particle irradiation. The drop was, however, more significant for EC-9323-2, with a decrease of almost 50%, while the value of EC-2216 dropped by only 5%. In contrast, after TVAC, the $G_{I_{c}}$ value for EC-9323-2 increased by 8%, and for EC-2216, the $G_{I_{c}}$ value increased by 12% on average. It is interesting to note that TVAC also increased the tested parameters (here, fracture toughness) of EC-2216 as it did for the tensile test values. TVAC also increased the $G_{I_{c}}$ values for EC-9323-2, but due to significant manufacturing defects in these adhesive samples, the results were less representative than EC-2216.

Concerning the failure type, for EC-2216, both aging processes increased the area of adhesive failure and therefore decreased the proportion of cohesive failure. This means that exposure to space environment-related conditions significantly deteriorated the adhesion to CFRP. For EC-9323-2, the area of adhesive failure also increased. However, samples manufactured with this adhesive had more manufacturing defects, which led to a significant adhesive failure even for the unaged samples.

**Figure 10:**
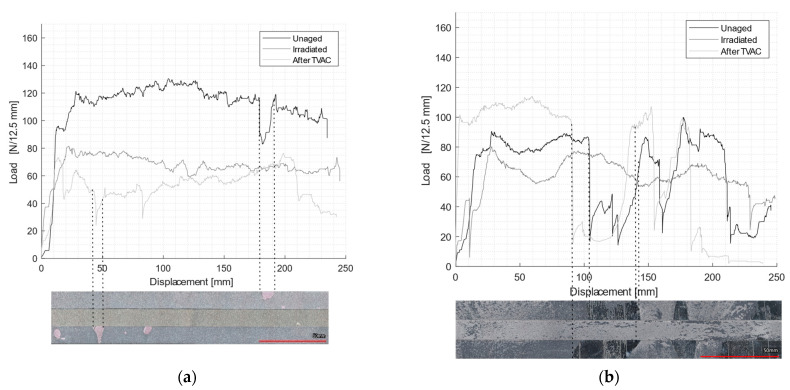
Load displacement curves for peel test sample: non-aged, after irradiation, and after TVAC for each adhesive; the correspondent failure surface of rigid adherent for (**a**) EC-2216 and (**b**) EC-9323-2.

The floating roller peel test load-displacement curves for unaged, irradiated, and post-TVAC conditions are reported in Figure 10. Pictures of the fracture surface of the rigid adherent are displayed below each load-displacement curve. As expected, there was an intimate relation between the peel load and the type of failure. For each of the two surface images, the top sample was the unaged sample, the middle one was the irradiated sample and the bottom one was the sample subjected to TVAC. In addition, the average peel load values and different failure mode proportions (CF, AF, and MD (manufacturing defect)) were collected and recorded in Table 5.

EC-2216 showed very few manufacturing defects and a failure of 97% cohesive for unaged samples, which shows the very good quality of the bond line for this adhesive. After the radiation of EC-2216, the average peel load decreased by 38% compared to the unaged profile, and after TVAC, the average peel load decreased by about 55%. Moreover, irradiation did not have a significant effect on the failure mode (93% cohesive failure); however, after TVAC, the adhesive failure increased significantly to approximately 30%. Although results from the DCB and tensile tests showed that after TVAC, for EC-2216, the mechanical properties increased, this did not hold true for the floating roller peel tests results, meaning the adhesion to aluminum was negatively affected.

**Figure 11:**
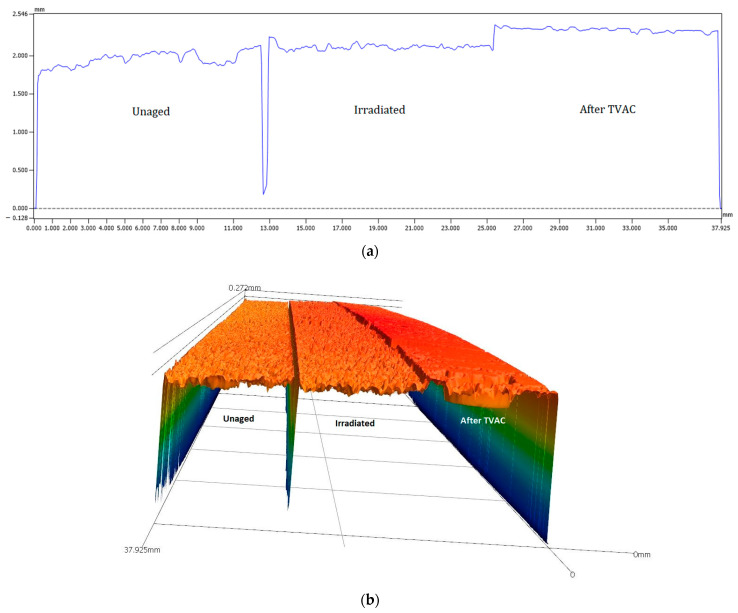
(**a**,**b**) 2D and 3D profiles of EC-2216 thickness present on rigid adherent after testing (unaged on the left, irradiated in the center, and TVAC on the right).

Figure 11 shows the 2D and 3D profiles of the rigid adherent of EC-2216, unaged, after irradiation, and after TVAC. It is shown that after TVAC, the adhesive thickness remaining on the rigid adherent part was much higher than for the other aging sample. This indicates that the fractured occurred closer to the interface in the case of TVAC than for the unaged and irradiation samples. Indeed, the surface of the specimen after TVAC (Figure 10) was slightly pink, which corresponds to the color of the primer applied to the aluminum during the surface treatment. This means that the crack propagated within the primer and not within adhesive. Peel load values after TVAC therefore not only represented the peel load created by the adhesive but also the peel load created by the primer. This value is still quite usable as it qualifies the ability of the sample to resist peeling after thermal aging in a vacuum.

A third failure mode must be considered for peel tests: manufacturing defect (MD). The latter was mainly present on EC-9323-2 specimens. It was represented through a very particular effect on the surface: each of the two faces had adhesive on it, which could suggest a cohesive failure at first sight, but these areas were shiny, which indicated that there had been no bonding between the two faces in this area. The defects were similar to those obtained in the DCB tests with the same adhesive. This reinforces the hypothesis that the entire process used for this adhesive was not optimized, especially in relation to the work life. It was witnessed that during the mixing, an exothermic reaction occurred, and that the curing reaction started before the bonding. Thus, the quality of the bond line was reduced.

## 4. Conclusions

This study aimed to assess the material properties and adhesion properties of EC-2216 and EC-9323-2 adhesives to CFRP and aluminum under space aging conditions, specifically thermal vacuum cycling and particle irradiation. Various tests, including tensile tests, DMA, DCB tests, and floating roller peel tests, were conducted to evaluate the effects.

For Scotch Weld EC-2216, neither electron irradiation nor thermal vacuum cycling had an impact on the glass transition temperature (T_g_). However, both conditions resulted in a decrease in peel strength, with reductions of 50% and 39% for samples subjected to thermal vacuum cycling and irradiation, respectively. The DCB test with CFRP adherents showed a slight increase of 12% in $G_{I_{c}}$ (fracture toughness) after thermal vacuum cycling but a small decrease of 5% after irradiation. Tensile properties were positively affected by thermal vacuum cycling, exhibiting a significant increase in modulus and tensile strength. However, a decrease in tensile properties was observed after irradiation. Such reduced performance after irradiation might be attributed to electron fluxes affecting the polymers through chain scissions and cross-linking [29], hence changing their mechanical characteristics and creating embrittlement. Indeed, a reduction in Young’s modulus, as observed for Scotch Weld EC-2216, might be explained by chain scission, with the small polymer chains being able to embed themselves between the large ones and plasticizing the adhesive. On the other hand, the increase in Young’s modulus observed in the TVAC tests can be explained by the cross-linking of the polymer chains.

Regarding Scotch Weld EC-9323-2, the T_g_ decreased after both thermal vacuum cycling and irradiation. In both cases, the tensile modulus was negatively affected, with a decrease of up to 50%. Thermal vacuum cycling had a positive effect on $G_{I_{c}}$, resulting in an 8% increase, as well as peel strength, with a 20% increase. However, significant negative effects were observed after irradiation, leading to a 50% decrease in $G_{I_{c}}$ and a 30% decrease in peel strength.

This study provides valuable insights into the macroscopic phenomena induced by space environment conditions. However, the results do not provide insights into the molecular behavior of adhesives under space aging conditions. Further detailed studies on these behaviors would be necessary to develop predictive models for the degradation of adhesive and adhesion properties under space aging conditions. Such models would enable the quantification of adhesive joint durability, allowing for lifetime predictions and facilitating the design of more robust and suitable structures for long-term missions.

## Figures and Tables

**Table 1:** Adhesive materials and corresponding curing cycle.

Adhesive	Curing Temperature (°C)	Curing Time (min)	Part A/B (Weight Ratio)
EC 2216	66	120	7/5
EA 9323-2	65	120	1/2

**Table 2:** Average tensile properties of EC-2216 and EC-9323-2, including standard deviation.

Aging	Scotch-Weld™ EC-2216			Scotch-Weld™ EC-9323-2		
	E (MPA)	σmax (MPA)	εmax	E (MPA)	σmax (MPA)	εmax
Unaged	242 ± 48	19 ± 1.3	0.74 ± 0.08	2197 ± 703	20 ± 4.2	0.027 ± 0.007
Irradiated	123 ± 49	15 ± 3.3	0.53 ± 0.29	1026 ± 267	17 ± 2.2	0.026 ± 0.008
After TVAC	606 ± 130	28 ± 1.5	0.61 ± 0.07	1042 ± 198	18 ± 1.0	0.033 ± 0.008

**Table 3:** Average glass temperature evolution as function of the environmental exposure.

Aging	T_g_ from Max Tan δ (°C)	
	Scotch-Weld™ EC-2216	Scotch-Weld™ EC-9323-2
Unaged	28 ± 1.1	91 ± 7.4
Irradiated	30 ± 2.2	72 ± 3.6
After TVAC	30 ± 1.8	68 ± 7.8

**Table 4:** Double-cantilever bean test result with evaluation of cohesive (CF) and adhesive (AF) failure mode.

Aging	Scotch-Weld™ EC-2216			Scotch-Weld™ EC-9323-2		
	$G_{I_{c}}$	Failure Mode		$G_{I_{c}}$	Failure Mode	
	(on CF)	CF (%)	AF (%)	(on CF)	CF (%)	AF (%)
Unaged	1203 ± 52	61 ± 15	39 ± 15	280 ± 100	10 ± 10	90 ± 11
Irradiated	1142 ± 79	41 ± 23	59 ± 23	142 ± 11	0.5 ± 1	100 ± 1
After TVAC	1348 ± 130	31 ± 13	69 ± 13	302 ± 33	0 ± 0	100 ± 0

**Table 5:** Average peel load and failure mode identification.

Aging	Scotch-Weld™ EC-2216				Scotch-Weld™ EC-9323-2			
	F_ave_ (N/12.5 mm)	Failure Mode			F_ave_ (N/12.5 mm)	Failure Mode		
		CF (%)	AF (%)	MD (%)		CF (%)	AF (%)	MD (%)
Unaged	111 ± 6.4	97 ± 2.2	0.8 ± 1.1	2 ± 1.3	87 ± 8	54 ± 27.2	0 ± 0	46 ± 27.2
Irradiated	68 ± 3.5	93 ± 6.1	6 ± 6.5	0.4 ± 0.8	62 ± 5.5	63 ± 23.9	2.2 ± 8	35 ± 26.2
After TVAC	53 ± 6	63 ± 17.7	30 ± 20	7 ± 1.4	105 ± 5	50 ± 7.3	0 ± 0	49 ± 7.3

## Data Availability

The data presented in this study are available on request from the corresponding author.
